# The effects of happiness and hope on executive functions

**DOI:** 10.3389/fpsyg.2025.1617975

**Published:** 2025-08-11

**Authors:** Franziska Lautenbach

**Affiliations:** ^1^Institute of Sport Science, Humboldt-Universität zu Berin, Berlin, Germany; ^2^Faculty of Sport Science, Leipzig University, Leipzig, Germany

**Keywords:** positive emotion, cognition, positive mood, positive affect, pride

## Abstract

The notion that positive emotions always yield positive outcomes is compelling, yet prior meta-analytic findings (19 effect sizes) suggest no impact on executive functions. Limitations have been noted regarding the induction of specific positive emotions and assessment quality, especially for cognitive flexibility and working memory. To expand on this, the current studies induced happiness and hope in college students to examine effects on inhibition, cognitive flexibility (study 1, *N* = 27), and working memory (study 2, *N* = 30). Results confirmed successful emotion induction and revealed that cognitive flexibility was significantly higher in the happiness condition than in a neutral condition (*p* = 0.014, *d* = 0.427). Findings suggest challenges in experimentally differentiating discrete positive emotions and indicate that not all executive functions are equally affected. Overall, these results lend support to Isen’s facilitator theory but should be interpreted with caution.

## Introduction

How positive emotions (PEs) can impact cognitive processes has been a growing interest over the past few decades; however, there is disagreement. There are theoretical models that depict a facilitating impact of PEs on cognition (e.g., facilitator theory), while others emphasize a negative influence (e.g., cognitive load theory). A recent meta-analysis concluded that PEs do not have a significant impact on executive functions (EFs: inhibition, cognitive flexibility, and working memory; Lautenbach, 2024). However, further investigations appear necessary, especially because the methods used to assess EFs varied between studies, and the assessment of cognitive flexibility and working memory is critical (Lautenbach, 2024). At the same time, most of the recent studies assessed valence as part of affect as the main indication for a successful emotion induction, and only a few studies actually compare PEs within a single study (e.g., Cameron et al., 2018). Thus, the aim of the current studies is to overcome some of the limitations and deepen our understanding of the impact of PEs on cognition, specifically on EFs. Therefore, in a within-subject design, happiness and hope were induced via false feedback, and all core EFs were assessed via well-known and reliable computer-based tasks (i.e., study 1: flanker task, number-letter task; study 2: *n*-back task).

### Positive emotions

PEs are “brief, multisystem responses” to positive appraisals of current circumstances (Fredrickson, 2013, p. 3–4). Different researchers categorize PEs uniquely. For example, Fredrickson lists joy, gratitude, serenity, and hope among the top 10 PEs, while Shiota et al. (2014) identify emotions such as pride, awe, and contentment in the PANACEAS (i.e., Pride, amusement, nurturant love, attachment love, contentment, enthusiasm, awe, and sexual desire) taxonomy. Lazarus (2000) classifies emotions such as compassion, happiness, and pride as PEs but considers hope and relief borderline cases, as they may include both positive and negative elements. A consensus on defining discrete PEs has not been reached, indicating a need for further research (Villanueva et al., 2021).

Happiness (or joy) and hope are widely recognized as distinct positive emotions (Fredrickson, 2013; Lazarus, 2000). Lazarus uses happiness and joy interchangeably, viewing it as progress toward a goal, while Fredrickson sees it as arising from unexpected good fortune. Lazarus’s definition of happiness emphasizes continuous goal pursuit and fostering sustained motivation, while Fredrickson describes joy as sparking an urge to play, which can be physically, socially, or intellectually, or engage without a specific aim. For this study, aspects of both definitions are incorporated to facilitate experimental manipulation. In detail, happiness will be defined in line with Lazarus’s goal-oriented perspective (i.e., participants are provided with an experimental task) and also in line with Fredrickson’s urge to play physically (i.e., physical activation due to experimental task) and later intellectually (i.e., cognitive task).

Hope is defined as “fearing the worst but yearning for better, and believing improvement is possible” (Lazarus, 2000, p. 234). It often emerges in difficult circumstances, motivating individuals to use their capabilities to change the situation (Fredrickson, 2013). Snyder describes hope as a cognitive orientation involving goal-directed determination (agency) and strategies for achieving goals (pathways; Snyder et al., 1996). Both definitions suggest that hope, similar to happiness, fosters strong motivation, supporting its role as a motivational state (Gustafsson et al., 2010). However, hope’s action tendency includes an individual to plan for the future and thereby, draw upon their own resources (Fredrickson, 2013).

Operationalizing PEs experimentally is challenging (e.g., Pourtois et al., 2017), and clear distinctions between them remain elusive (Roth and Laireiter, 2021). This may explain why most studies have focused on general positive affect or mood, while only about one in five have experimentally induced specific PEs, that is, happiness or joy (see review by Joseph et al., 2020; Lautenbach, 2024), predominantly using film clips (Lench et al., 2011). However, this approach is not equally relevant for all target groups and does not necessarily capture the context-dependent nature of emotional experiences. In other words, positive emotions such as happiness or joy do not naturally arise solely from passively watching films but are more commonly elicited through social interaction, play (Fredrickson, 1998), or successful task performance (Lazarus, 2000). To enhance the ecological validity of PE inductions, more contextually appropriate methods should be employed. Therefore, the current study aim to incorporate a physical performance-related task for sport students, as previous research has demonstrated positive correlations between PEs and athletic performance (Moen et al., 2018).

In addition, when assessing affective or emotional states, only one in five studies employs standardized questionnaires for distinct emotions, while the majority rely on broader affect measurements, such as valence (Lautenbach, 2024). Therefore, the current studies aim to use questionnaires to assess discrete emotions.

Finally, not only do studies rarely compare positive emotions to neutral conditions (approximately one-third; see Lench et al., 2011, p. 843), but none of the studies analyzed by Lench et al. (2011) directly compared discrete positive emotions with one another. Altogether, only a few studies have done so (e.g., Cameron et al., 2018), which is problematic as different PEs may affect cognitive processes differently (Griskevicius et al., 2010). In detail, based on the provided definitions and theoretical perspectives on the cognitive effects of happiness and hope (see below), one could argue that happiness is more likely to broaden the thought-action repertoire (Fredrickson, 1998). However, this broadening may come at the cost of less detailed processing of incoming information, potentially leading to decreased cognitive performance (Griskevicius et al., 2010). In contrast, hope incorporates an element of fear, which individuals seek to minimize by drawing on their own resources and planning for the future (Fredrickson, 2013). Consequently, hope may promote a more detailed processing of information and enhance cognitive performance. This theoretical distinction highlights that PE is not a single, uniform construct but rather consists of distinct emotional states, each with unique functional effects that warrant individual investigation (Griskevicius et al., 2010). Thus, this study aims to induce happiness and hope as discrete emotions to explore their distinct effects on cognitive processes, particularly executive functions.

### Executive functions

EFs are specific cognitive processes that support attention and goal-directed behavior (Lautenbach et al., 2024). EFs play a key role in mental and physical health, school achievement, career success, and public safety (Diamond, 2013). Core EFs include inhibitory control (overriding internal or external impulses), cognitive flexibility (adapting to new demands or shifting tasks), and working memory (holding and manipulating information; Diamond, 2013). Higher-level EFs, such as reasoning, planning, and problem-solving, depend on these core processes (Diamond, 2013).

Recently, the assessment of core EFs, especially cognitive flexibility and working memory, has been criticized (Lautenbach, 2024). Whereas inhibition is commonly assessed with standardized tasks, such as the flanker or Stroop tasks, cognitive flexibility was often measured using non-standardized tasks, such as word naming. For working memory, only two of four studies used the standardized *n*-back task (Au and Tang, 2019; Guo et al., 2020), while the others used memory tasks that did not require mental working with information (Lautenbach, 2024). Furthermore, increasing the number of trials is recommended to reduce variance (Lautenbach, 2024). Therefore, this study will use standardized computer tasks with an adequate number of trials to measure inhibition (flanker task), cognitive flexibility (number-letter task), and working memory (*n*-back task).

### Theories on positive emotions impacting cognition

Theoretical models on the effects of PEs on cognition, particularly EFs, suggest either facilitation or hindrance (Lautenbach et al., 2024; Mitchell and Phillips, 2007). The flexibility hypothesis proposes that PEs broaden focus while maintaining attention to details, enhancing inhibition and cognitive flexibility (Isen, 2009). The dopamine hypothesis (Ashby et al., 1999) links positive affect with increased dopamine levels, facilitating working memory and attention. Fredrickson’s broaden-and-build theory (2001) suggests that PEs expand thought-action repertoires and build personal resources. While specific predictions are challenging (Revord et al., 2021), it is likely that cognitive flexibility increases, while inhibition, requiring close attention, might decrease.

Contrary to theories suggesting positive impacts of PEs on cognition (e.g., Isen, Ashby, and Fredrickson), capacity theories, such as cognitive load theory (Sweller et al., 1998), argue that emotions consume cognitive resources by activating emotion-related networks, impairing cognitive performance through heuristic processing (Mitchell and Phillips, 2007). Similarly, the mood-as-information theory suggests that positive emotions reduce perceived threats, leading to less rigorous problem-solving and more reliance on heuristics. Since EFs require significant attentional control (Miyake and Friedman, 2012), this could result in decreased cognitive performance.

### Empirical findings on the effects of positive emotion on cognition

There are several previous reviews that have tried to support the theoretical approaches focusing on the impact of PEs on cognition with empirical evidence, and the results are mixed (e.g., the review by Mitchell and Phillips, 2007), with some even reporting no effects (see the review and meta-analysis by Lautenbach, 2024).

Studies on the impact of PEs on inhibition show mixed results. Some experiments (Rowe et al., 2007; Phillips et al., 2002) found impairment, while others (Wenzel et al., 2013) observed increased inhibitory control. All studies used similar participant structures (age and gender balance) and standardized tasks (flanker and Stroop) with sufficient trials. However, while positive affect was increased, discrete emotions were not assessed. The contradictory findings highlight the need for further empirical evidence to better understand the effect of PEs on inhibition, a key aspect of executive functioning (Miyake and Friedman, 2012).

No consistent effect of PEs on cognitive flexibility has been found, despite its importance for creativity (Diamond, 2013). While some studies show a positive impact (e.g., De Dreu et al., 2008), others do not. Future studies should use tasks such as switching paradigms to better assess adaptation to changing rules (Lautenbach, 2024). This study aims to implement more rigorous computer-based measures of cognitive flexibility.

Finally, results on working memory performance show mixed findings (Lautenbach, 2024). Four meta-analysis experiments revealed no effect in two studies (Martin and Kerns, 2010; Guo et al., 2020), while one study found an increase (Au and Tang, 2019) and another a decrease (Martin and Kerns, 2010). Despite using similar affect inductions (e.g., films), the studies had different designs and insufficient trials (13–18 trials). To address this, the present study will increase the number of trials.

### The present study

Research on the effects of PEs on EFs remains inconclusive, most likely due to methodological differences (i.e., sample and assessment of PEs and EFs; see Mitchell and Phillips, 2007; Lautenbach, 2024; Revord et al., 2021). To address this, the current study will use standardized computer tasks with more trials to reduce variance. Two discrete PEs, happiness and hope, will be induced through false feedback on a performance task, targeting sports students engaged in regular physical activity. Previous studies have shown positive correlations between these emotions and athletic performance (Moen et al., 2018).

First, it is hypothesized that happiness will increase in the happiness condition compared to the neutral condition (hypothesis 1a) and hope will increase in the hope condition compared to the neutral condition (hypothesis 1b, based on Lazarus’ core themes). It is expected that inhibitory performance (flanker effect response times and accuracy) will not differ between the PE and control conditions (hypothesis 2a, based on meta-analysis by Lautenbach, 2024). However, cognitive flexibility (switch cost, response times, and accuracy) will be better in the PE conditions compared to the control condition (hypothesis 2b, based on Isen’s flexibility hypothesis, as well as insufficient data in Lautenbach, 2024). No specific hypothesis is made regarding differences between happiness and hope conditions, even though a theoretical distinction was presented. Finally, working memory performance (response times and accuracy) will be better in the PE conditions compared to the control condition (hypothesis 2c, based on Au and Tang, 2019, as well as insufficient data in Lautenbach, 2024). No specific hypothesis is made about differences between the happiness and hope conditions, even though a theoretical distinction was presented.

## Methods

### Participants

*A priori* power analysis was conducted using G-Power analysis (Faul et al., 2007). A repeated measure, within-subject MANOVA using the effect size of η^2^ = 0.11 in Woodman et al. (2009) and a corrected alpha error probability (*α* = 0.025) to account for multiple dependent variables (Tabachnick et al., 2013), resulted in 36 participants (1 − *β* = 0.95).

Participants were sports students who were recruited during the semester in a seminar on sport psychology. They received no financial reward. The study protocol, including an informed consent form and debriefing information, was approved by the ethics committee of the local university (#2019.03.08_eb_7) and was conducted in accordance with the Declaration of Helsinki.

Two studies have been conducted: In study 1, assessing inhibition and cognitive flexibility, only data of 27 participants (*M_age_* = 21.07, *SD* = 1.54; 14 men, 13 women; all Caucasian) could be analyzed due to missing data (*n* = 2) or suspicion about the cover story (*n* = 8).

In study 2, assessing working memory, only data of 30 participants (*M_age_* = 21.67, *SD* = 4.60; 14 men, 16 women; all Caucasian) could be analyzed due to missing data (*n* = 3) or suspicion about the cover story (*n* = 3).

### Material

#### Cover story

Participants were told that the experiment was about the relationship between *playing* and cognitive performance. They were told that they are in the “*wobble board*” group and that testing repetitively (i.e., three times) is necessary to find stable effects. They were also told that they had to step on a *laboratory version* of the wobble board (i.e., Posturomed) to control that they actually try to perform their best during the game.

#### Positive emotion inductions

Participants had three trials each, with four trials in the hope condition on the Posturomed. They received verbal false feedback after each trial that was based on Lazarus’ core relation themes (2000). In addition, after the last trial, they were asked to use the experimenter’s laptop to see their performance visualized. This step was taken to reinforce the emotional induction as well as to maintain the cover story. Please refer to Table 1 for the detailed verbal and visual feedback.

**Table 1:** False feedback for the participants in each condition.

		Happiness condition	Hope condition	Neutral condition
Verbal feedback	After 1^st^ trial	This was really good. Let‘s see if you can be better than this.	This was really good. Let‘s see this time whether you can keep this when we increase the level of difficulty.	Thank you. Next round.
	After 2^nd^ trial	You’re really were better than last time. Let‘s see if you can keep this up for the next round.	Ohhh, you were almost as good but still not quite. Let‘s try again, I have hope.	Thank you. Next round.
	After 3^rd^ trial	Your last round was even better than the first and the second.	Ohhh, almost, so close. You were almost as good. For the next round just focus on one point, I am sure you will be as good as during your first try or even better. I have hope.	Thank you. Next round.
Visual feedback after 3^rd^ trial		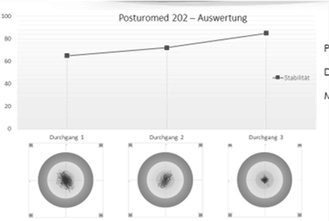	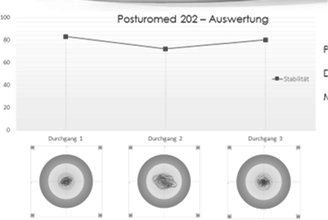	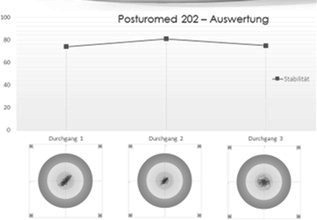

#### Emotions

Happiness. The subscale *happiness* from the Sport Emotion Questionnaire (Jones et al., 2005) was used to assess the discrete emotion happiness. Participants were asked to answer how they feel at the current moment on a 5-point Likert scale, ranging from 0 = *not at all* to 4 = *extremely*. The items were pleased, joyful, happy, and cheerful. The reliability of the subscale within our sample can be considered good (study 1 in neutral pre-condition: α = 0.807; study 2 in neutral pre-condition: *α* = 0.865).

Hope. The State Hope Scale (Snyder et al., 1996) was used to assess the discrete emotion hope. On two subscales (agency: “Right now I see myself as being pretty successful.”; pathways: “I can think of many ways to reach my current goals.”) with three items each, participants were asked to respond on an 8-point Likert scale, ranging from 1 = *definitely false* to 8 = *definitely true*. The reliability within our sample can be considered good (study 1 in neutral pre-condition agency: α = 0.872; pathways: α = 0.886, study 2 in neutral pre-condition agency: α = 0.790; pathways: α = 0.753).

### Executive functions

All tasks were measured on a 15-in. flat-screen monitor (1,280 × 960 pixels at 60 Hz) at a viewing distance of approximately 60 cm, using Inquisit 5 (2018). All responses were provided via button press on a QWERTZ keyboard.

Inhibition. The arrow flanker task was used to assess inhibition (Eriksen and Eriksen, 1974). In total, five black arrows are presented on a white background. Participants are asked to respond as quickly and correctly as possible to which direction the middle arrow is pointing (left: press “E”; right: press “I”). In congruent trials, all arrows (i.e., the target arrow and flanker arrows) are pointing in the same direction, whereas in incongruent trials, the target arrow is pointing in a different direction, making it more difficult to inhibit the flanker arrows and thus leading to the flanker effect. The flanker effect is the difference between the response times for incongruent trials and the response times for congruent trials. A lower flanker effect represents better inhibitory control.

In total, participants performed four practice trials in which 75% had to be answered correctly. This was followed by two blocks of 72 trials. We implemented twice as many congruent trials (i.e., per block 48) in comparison to incongruent trials (i.e., per block 24), as it has been argued to warrant higher demands on inhibitory control (e.g., Musculus et al., 2022).

Cognitive Flexibility. The number-letter task was used to assess cognitive flexibility (adapted from Rogers and Monsell, 1995 by Miyake et al., 2000). Participants see a 2 × 2 matrix in which number-letter pairs appear in the quadrants. They are asked to respond as quickly and correctly as possible to either the presented number (bottom two quadrants: press “E” for even numbers; “I” for odd numbers) or the presented letter (top two quadrants: press “E” for a consonant; “I” for a vowel). Response time and accuracy are assessed for so-called non-switch trials (i.e., same task to either focus on number or letter) and switch trials (i.e., task change from focusing on number to focusing on letter and vice versa). The lower the switch cost, which is the difference between the response times for switch trials minus the response times for non-switch trials, the better the cognitive flexibility performance.

In total, participants performed 24 practice trials for the letter task, 24 practice trials for the number task, and 28 practice trials for the combined task in which they had to answer 75% correctly. This was followed by four blocks of 32 trials (128 trials in total: 64 switch trials, 64 non-switch trials).

Working Memory. The *n*-back task was used to assess working memory performance (Jaeggi et al., 2010). Participants are presented neutral pictures and have to decide as quickly and correctly as possible whether the same picture was presented *n* pictures back.

In total, participants performed 10 practice trials per level (2-back, 3-back, and 4-back) followed by three times three blocks, including a total of 66 2-back trials, 69 3-back trials, and 72 4-back trials. Each block included 18 target trials and 42 non-target trial, presenting a 30 to 70 ratio for target to non-target trials (e.g., Knöbel and Lautenbach, 2023). After each block, there was a short break.

### Procedure

**Figure 1:**
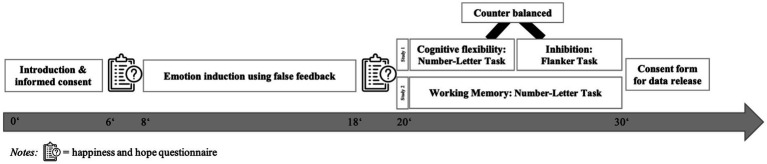
Detailed experimental procedure.

The experimental procedure took place in the same laboratory for both studies, with testing between 10 a.m. and 6 p.m. Each session lasted approximately 30 min, and participants completed three sessions, one per condition (neutral, happy, and hope) in a balanced order (Figure 1 for the procedure). To prevent fatigue due to differing task lengths, especially the working memory task, two separate studies were conducted.

Participants were welcomed and guided through the procedure via pre-recorded audio during the first session. They then signed consent and data protection forms and reported their current happiness and hope levels using questionnaires. After the emotion induction (happy, hope, or neutral), participants completed post-induction happiness and hope assessments. In study 1, inhibitory performance was measured with the flanker task, and cognitive flexibility with the number-letter task, both counterbalanced. In study 2, the n-back task was used for working memory. An additional Posturomed trial was included in the hope condition to maintain the cover story. Afterward, participants were debriefed and asked to sign a consent form for data release.

### Data reduction and data analyses

In study 1 (inhibition and cognitive flexibility), two participants had to be excluded from the analyses due to incomplete data (e.g., one condition was missing). Furthermore, eight participants were excluded as they expressed suspicion with regard to the cover story (e.g., “I think the feedback was fake.”). In study 2 (working memory), three participants had to be excluded from the analyses due to incomplete data. Furthermore, three participants were excluded as they expressed suspicion with regard to the cover story.

Data were further checked for normal distribution and outliers. In study 1, as well as in study 2, not all variables were normally distributed. However, based on the central limit theorem (Tavakoli, 2012) and the relative robustness of multivariate analyses of variance against violations of the normal distribution (Wilcox, 2011), parametric testing was performed. In study 1, one outlier (i.e., #20: neutral condition, flanker task, reaction time congruent trials, and incongruent trials) and in study 2, three outliers (i.e., #17: neutral condition, 2-back, reaction time; happy condition, 2-back, accuracy; #29: happy condition, 2-back, accuracy; #33: happy condition, 2-back, accuracy) were detected. However, the patterns of results were similar, and thus the results were reported, including the outlier.

To test hypotheses 1a and 1b (manipulation check) for both studies, 2 (time: pre vs. post) x 3 (condition: happy vs. hope vs. neutral) MANOVAs were ran, including the total score of happiness and hope. To test hypotheses 2a to 2c (effects on cognition), 2 (time: pre vs. post) x 3 (condition: happy vs. hope vs. neutral) MANOVAs for inhibition (reaction time and accuracy for congruent trials, incongruent trials, and flanker effect); cognitive flexibility (reaction time for no-switch trials, switch trials, and switch coasts; overall accuracy); and working memory (accuracy and reaction time for 2-back, 3-back, and 4-back) were performed. Significant effects were followed up by univariate testing, *post-hoc* analyses with Bonferroni corrections, and potential interaction effects with paired *t*-tests.

### Transparency and openness

The determination of the sample size, data exclusion, all manipulations, and all measures in the study are reported. All data, analysis code, and research materials are available upon request. This study’s’ design and its analysis were not pre-registered.

## Results

Please find the descriptive data for studies 1 and 2 on PEs in Table 2 and on EFs in Table 3.

**Table 2:** Descriptive data of happiness and hope in all conditions of study 1 and study 2.

		Pre *M* (*SD*)			Post *M* (*SD*)		
		Neutral condition	Happiness condition	Hope condition	Neutral condition	Happiness condition	Hope condition
Study 1	Happiness	2.56 (0.53)	2.31 (0.63)	2.36 (0.67)	2.61 (0.56)	2.71 (0.56)	2.69 (0.61)
	Hope	35.48(6.98)	34.48 (7.52)	34.00 (7.59)	35.56 (7.61)	36.67 (6.97)	35.67 (7.35)
Study 2	Happiness	2.33 (0.70)	2.17 (0.69)	2.33 (0.72)	2.38 (0.85)	2.48 (0.76)	2.57 (0.68)
	Hope	33.60(6.16)	32.97 (6.70)	32.63 (6.12)	33.33 (5.94)	34.50 (6.19)	34.67 (6.63)

N_study1_ = 27; N_study2_ = 30.

**Table 3:** Descriptive data for inhibition (flanker task), cognitive flexibility (number-letter task), and working memory (n-back task).

			Neutral *M* (*SD*)	Happiness *M* (*SD*)	Hope *M* (*SD*)
Inhibitory performance (study 1)	Congruent trials	Accuracy (in %)	98.65 (2.02)	99.07 (1.44)	99.97 (1.17)
		Reaction time (in ms)	381.72 (52.90)	375.68 (40.23)	379.86 (39.43)
	Incongruent trials	Accuracy (in %)	97.08 (2.71)	97.46 (2.90)	97.22 (3.37)
		Reaction time (in ms)	409.98 (60.29)	403.01 (44.22)	407.70 (42.73)
	Flanker effect	Reaction time (in ms)	28.26 (16.37)	27.33 (13.65)	27.84 (17.93)
Cognitive flexibility performance (study 1)		No switch trials (in ms)	764.89 (160.77)	786.25 (187.37)	775.26 (157.19)
		Switch trials (in ms)	1236.95 (410.93)	1158.14 (321.74)	1195.27 (321.18)
		Switch costs (in ms)	427.06 (274.99)	371.89 (185.67)	420.01 (227.42)
		Excluded trials (in total)	8.48 (4.94)	8.96 (6.78)	8.70 (5.11)
Working memory performance (study 2)	*2*-back	Accuracy (in %)	86.34 (14.09)	83.54 (16.95)	85.69 (13.33)
		Reaction time (in ms)	645.16 (170.38)	637.71 (180.70)	662.26 (179.39)
	*3*-back	Accuracy (in %)	51.24 (26.40)	55.80 (22.22)	57.51 (19.79)
		Reaction time (in ms)	821.33 (302.59)	831.59 (275.98)	773.24 (230.29)
	*4*-back	Accuracy (in %)	42.59 (13.35)	42.47 (20.64)	45.56 (20.50)
		Reaction time (in ms)	827.60 (320.03)	823.61 (310.68)	790.30 (317.29)

N_study1_ = 27; N_study2_ = 30.

### Study 1 and 2: Manipulation check (hypothesis 1a and 1b)

For study 1 (inhibition and cognitive flexibility), the MANOVA showed no main effect for condition (*p* = 0.810). However, a significant main effect for time, *F*(2, 25) = 10.68, *p* < 0.001, η_p_^2^ = 0.461, as well as a significant interaction effect, *F*(4, 102) = 5.02, *p* < 0.001, η_p_^2^ = 0.164, was detected, indicating a change of emotions depending on the condition. Univariate testing confirmed this effect for happiness (*p* < 0.001, η_p_^2^ = 0.267) and hope (*p* = 0.010, η_p_^2^ = 0.170). Paired *t*-tests showed that happiness was increased in the happiness, *t*(26) = 4.06, *p* < 0.001, *d* = 0.671, as well as in the hope condition, *t*(26) = 4.70, *p* < 0.001, *d* = 0.501. Similarly, hope was significantly increased in the happiness, *t*(26) = 3.19, *p* = 0.002, *d* = 0.302, and hope condition, *t*(26) = 3.93, *p* < 0.001, *d* = 0.223. No significant emotional changes (happiness: *p* = 0.141, hope: *p* = 0.441) were induced in the neutral condition.

For study 2 (working memory), the MANOVA showed no main effect for condition (*p* = 0.405). However, a significant main effect for time, *F*(2, 28) = 7.55, *p* = 0.002, η_p_^2^ = 0.350, as well as a significant interaction effect, *F*(4, 114) = 4.01, *p* = 0.004, η_p_^2^ = 0.123, was detected, indicating a change of emotions depending on the condition. Univariate testing confirmed this effect for happiness (*p* = 0.021, η_p_^2^ = 0.137) and hope (*p* = 0.010, η_p_^2^ = 0.159). Paired *t*-tests showed that happiness was increased in the happiness, *t*(29) = 4.43, *p* < 0.001, *d* = 0.426, as well as in the hope condition, *t*(29) = 3.48, *p* < 0.001, *d* = 0.346. Similarly, hope was significantly increased in the happiness, *t*(29) = 3.98, *p* < 0.001, *d* = 0.237, and hope condition, *t*(29) = 2.61, *p* = 0.007, *d* = 0.320. No significant emotional changes (happiness: *p* = 0.313, hope: *p* = 0.267) were induced in the neutral condition.

### Study 1: Effects of positive emotion on inhibition (Hypothesis 2a)

The MANOVA showed no significant effect for condition, *F*(10, 96) = 0.34, *p* = 0.968, η_p_^2^ = 0.034.

### Study 1: Effects of positive emotion on cognitive flexibility (Hypothesis 2b)

The MANOVA showed no significant effect for condition, *F*(6, 21) = 2.44, *p* = 0.060, η_p_^2^ = 0.411. However, as *p* is close to the level of significance, and to further scrutinize the results, univariate testing was inspected (e.g., Lautenbach et al., 2016). Only switch costs came close to significance (*p* = 0.060, η_p_^2^ = 0.096) and were further scrutinized using paired *t*-tests. No significant differences between the neutral and hope conditions (*p* = 0.133, *d* = 0.206), as well as between the hope and happiness conditions, were shown (*p* = 0.113, *d* = 0.232). However, in the happiness condition, participants had significantly lower switch costs in comparison to the neutral condition, *t*(26) = 2.31, *p* = 0.014, *d* = 0.427, indicating higher cognitive flexibility.

### Study 2: Effects of positive emotion on working memory (Hypothesis 2c)

The MANOVA showed no significant effect for condition, *F*(4, 116) = 0.71, *p* = 0.587, η_p_^2^ = 0.061. The significant main effect of *n*-backs, *F*(4, 114) = 43.96, *p* < 0.001, η_p_^2^ = 0.61, is also present for accuracy (*p* < 0.001, η_p_^2^ = 0.83) and reaction time (*p* < 0.001, η_p_^2^ = 0.36) and simply indicates that accuracy and reaction time are the best in the 2-back condition, followed by the 3-back, and finally the 4-back condition. More importantly, no interaction effect between *n*-backs and condition was detected, *F*(8, 230) = 0.72, *p* = 0.677, η_p_^2^ = 0.024.

## Discussion

The studies aimed to examine the impact of happiness and hope, induced by false feedback, on EFs (inhibition, cognitive flexibility, and working memory) using standardized tasks. While the emotions were successfully induced, they were not distinct, with happiness also increasing in the hope condition and vice versa. As a result, the effects on EFs need to be critically interpreted with caution. However, higher cognitive flexibility was observed in the happy condition compared to the neutral condition.

### Challenges to successfully induce discrete emotions

The results show that positive emotions were successfully induced in both the happy and hope conditions. However, both emotions increased in each condition, which may stem from a combination of theoretical and methodological factors.

Happiness and hope are closely linked emotions, as described in the OCC model (Ortony et al., 1988) and its revision (Steunebrink et al., 2009). Both are event-related emotions, with joy representing the pleasure of an actual event and hope arising from the possibility of a future event. Happiness typically results from desirable events, while hope emerges when outcomes are not yet realized. In the current study, the hope condition likely also induced happiness due to improved performance. Additionally, hope can foster emotional orientations, such as happiness, that support goal attainment (Oettingen and Gollwitzer, 2002). This overlap makes it difficult to distinguish between the two emotions, as demonstrated by Cameron et al. (2018), who found no difference between happiness and hope conditions. This highlights the challenge of inducing distinct positive emotions, especially when they share similar valence and arousal levels (Lautenbach, 2024; Lindquist et al., 2013).

This theoretical issue is mirrored by methodological challenges. The emotion induction for the hope condition was based on Lazarus’s core relational theme of “fearing for the worst, hoping for the best” (Lazarus, 2000, p. 234). However, it is questionable whether hope, as defined by Lazarus, was accurately captured, as participants did not face significant fears, and this aspect was not emphasized. Additionally, the hope questionnaire used was based on Snyder’s definition, where hope is “a cognitive set based on a reciprocally-derived sense of successful agency (goal-directed determination) and pathways (planning to meet goals)” (Snyder et al., 1996, p. 571). This conceptualization positions hope as a cognitive, goal-setting construct (see Oettingen and Gollwitzer, 2002), aligning more with motivation or self-efficacy theories (Gustafsson et al., 2010) than with Lazarus’s definition. Thus, the theoretical framework and the methodological assessment of hope differ significantly.

Finally, methodological challenges (e.g., task design and experimenter influence) may have hindered differentiation between the two positive emotion conditions. The active performance task aimed to elevate positive emotions in athletes, as athletic performance correlates with positive emotions (Moen et al., 2018). However, this task can inherently motivate participants (Trecroci et al., 2018; Volery et al., 2017) and in addition elicit emotional responses (Quigley et al., 2014). While positive affect increases with success, it is unclear how to design a task to induce distinct emotional states (Nummenmaa and Niemi, 2004). Since no changes were observed in the neutral condition and affective reactions to performance tasks—especially with feedback—are influenced by social perception, it is likely that the experimenter’s social component played a significant role (Nummenmaa and Niemi, 2004).

The involvement of the experimenter could introduce complications. While the experimenter used different wording in each condition, they maintained similar non-verbal behavior, speaking in a friendly tone and showing attentiveness. This method, resembling emotion induction through confederates (Quigley et al., 2014), presents challenges as confederates or experimenters must be convincing and standardize details like tone, intonation, and non-verbal communication (Quigley et al., 2014). Emotional contagion (Hatfield et al., 1993) may have also occurred, unintentionally triggering emotional states. Given that the experimenter conducted up to eight tests daily under varying emotional conditions, their own mood and consistent feedback may have influenced emotional intensity. A second experimenter providing additional feedback or an emotion booster in study 1 between the two EF tasks (e.g., for stress Angelidis et al., 2019) could have heightened and/or maintained emotional intensity (Shteynberg et al., 2014). Overall, the absence of professional actors may have contributed to the difficulty in inducing discrete emotions.

### Impact of PEs on EFs

Despite challenges in inducing discrete emotions, there was a measurable increase in both happiness and hope across the positive emotion conditions. While happiness and hope values in the post-measurement were comparable across all conditions, the observed increases in positive emotions may have influenced cognitive performance. In other words, changing emotional state, even without lasting emotional differences, might have a minimal impact on cognitive performance (Durlak et al., 2011). Although speculative in this study’s context, this argument is supported by prior research that found no correlations between positive emotions in neutral states and cognitive flexibility (Knöbel et al., 2024).

The hypothesis that an increase in happiness—or positive emotions more broadly—might facilitate cognitive flexibility received some support, particularly with regard to the main performance indicator, switch cost. Although a recent meta-analysis (Lautenbach, 2024) found inconsistent effects of positive emotions on cognitive flexibility, it included only five studies with highly heterogeneous effect sizes and critically evaluated measurement methods. While the present findings differ from the meta-analysis, they align with the theoretical framework that suggests positive emotions enhance creativity (the flexibility hypothesis, Isen, 2009), where cognitive flexibility plays a key role (Diamond, 2013). Prior research has also demonstrated a positive impact of positive emotions on creativity (Baas et al., 2008), supporting the current findings.

The lack of influence on other executive functions aligns with the hypothesis for inhibition (hypothesis 2a) but contradicts expectations for working memory (hypothesis 2c). Inhibitory performance remained unaffected by emotional changes. One possible explanation is that positive emotions reduce the perceived difficulty of the task (Grahek et al., 2020). Since the flanker task is relatively simple, it may be perceived as even easier in the presence of increased positive emotions (see also Lautenbach, 2024). Future studies should measure and control for task difficulty in EF tasks.

Contrary to expectations, no effect on working memory was found. This aligns with Lautenbach (2024) meta-analysis, which was based on a small number of studies (*n* = 4) with significant variability in effect sizes (*d* = −0.32 to 0.667), limiting its validity. Only one study (Au and Tang, 2019) used the *n*-back task with fewer trials than the current study, suggesting their findings may have been coincidental. Future research should further explore the impact of positive emotions on working memory.

### Limitation

Both studies have limitations that should be addressed in future research, with the primary challenge being the induction of discrete PEs (Roth and Laireiter, 2021) and its measurements. Few studies have successfully induced different discrete emotions, and many attempts have been unsuccessful (e.g., Cameron et al., 2018). Thus, the current results align with previous research, but the issue of inducing discrete PEs remains unresolved. Thus, further research is needed to better differentiate PEs and develop effective and ecologically valid induction methods (Pourtois et al., 2017). A more feasible approach to identifying effects on desired dependent variables may also be to induce a single positive emotion and compare it with a neutral or negative emotion to clarify its effects on EFs (Lautenbach, 2024).

However, this approach may inadvertently obscure relevant aspects of emotional experience. Experimental manipulations rarely elicit a single, isolated emotion; rather, it is both possible and psychologically normative to experience multiple emotions simultaneously (Carrera and Oceja, 2007). In the present context, it appears entirely plausible that—beyond for example the target emotion happiness—additional positive emotions, such as pride (see definition by Fredrickson, 2013), were elicited, given that individuals received social acknowledgment for their (even minor) achievements (i.e., increase in performance). In addition, the notion that happiness and pride often co-occur—though distinct in their antecedents—has been particularly emphasized in performance contexts, with both emotions serving as clear motivational drivers to performance (Lazarus, 2000).

These considerations highlight a broader methodological concern—one that is compellingly exemplified by the present data: the necessity of critically reflecting on how we conceptualize and measure emotions. If, for example, we had employed only one experimental condition and exclusively measured happiness, the resulting data might have appeared clear-cut and compelling. However, such clarity would have come at the cost of ignoring the broader emotional landscape, thereby offering a narrow and potentially misleading representation of the actual affective experience. This concern is echoed in the literature. It has been emphasized that measurements never fully capture the complexity of psychological reality, but rather represent only a selective excerpt shaped by the way constructs are operationalized (e.g., Shadish et al., 2002). In addition, the current study operationalized emotions solely through subjective experience, thereby leaving other relevant components of emotional responding—crucial for a comprehensive understanding of emotional processes—unexamined (see review by Mauss and Robinson, 2009). Moreover, factors such as personality and emotion regulation processes—which have been associated with executive functions (see meta-analysis by Toh et al., 2024)—may also modulate the impact of emotion induction.

Further methodological and statistical challenges may have contributed to the indistinct effects of PEs observed. The complex design, including an elaborate cover story, led to participant exclusions due to missing data (*n* = 5) or doubts about the cover story (*n* = 11), with 15.07% expressing doubt, consistent with previous findings (Rahwan et al., 2022). This reduced sample size impacted statistical power. *Post-hoc* power analysis showed power for inducing positive emotions was 0.30 in Study 1 and 0.22 in Study 2, and for detecting effects on EFs, power was 0.09 for inhibition, 0.91 for cognitive flexibility, and 0.15 for working memory. A larger sample would have improved statistical power, and future studies should aim for 10–20% more participants. Due to substantial constraints—particularly limited time resources, and insufficient personnel capacity—oversampling was unfortunately not feasible in the current research context. Despite this, the sample size was sufficient to detect an effect on cognitive flexibility.

Finally, sample characteristics such as age and gender may play significant roles. For instance, Grossman and Wood (1997) found that women responded more strongly to emotional inductions of happiness, and older adults tend to focus more on positive emotions (Tsai et al., 2000). However, *post-hoc* analyses controlling for gender and examining age correlations did not yield additional relevant findings. Future studies should continue to control for participant characteristics to better understand their potential influences.

## Conclusion

Positive emotions are common in daily life (Li et al., 2020) and integral to human experience (Fredrickson, 2013), while executive functions are critical for higher-order cognitive processes and impact various life aspects, including academic success (Diamond, 2013). The influence of positive emotions on executive functions warrants further investigation, as current studies suggest these emotions may have some sort of measurable impact. Future research should prioritize the differentiated induction of discrete positive emotions and focus on higher-order executive functions, which are relevant to real-life situations. If positive emotions are shown to enhance higher-order executive functions, this could inform interventions to improve cognitive functioning, such as in educational contexts to boost student engagement and learning outcomes. Additionally, focusing on higher-order executive functions can refine theoretical models of emotional–cognitive interactions, offering a more nuanced understanding of how emotions influence cognitive processes.

## Data Availability

The raw data supporting the conclusions of this article will be made available by the authors, without undue reservation.
